# Eosinophilic Inflammation in Allergic Asthma

**DOI:** 10.3389/fphar.2013.00046

**Published:** 2013-04-17

**Authors:** Samantha S. Possa, Edna A. Leick, Carla M. Prado, Mílton A. Martins, Iolanda F. L. C. Tibério

**Affiliations:** ^1^Department of Medicine, School of Medicine, University of São PauloSão Paulo, Brazil; ^2^Department of Biological Science, Federal University of São PauloSão Paulo, Brazil

**Keywords:** airway remodeling, asthma, eosinophils, experimental models of asthma, inflammation, respiratory hypersensitivity

## Abstract

Eosinophils are circulating granulocytes involved in pathogenesis of asthma. A cascade of processes directed by Th2 cytokine producing T-cells influence the recruitment of eosinophils into the lungs. Furthermore, multiple elements including interleukin (IL)-5, IL-13, chemoattractants such as eotaxin, Clara cells, and CC chemokine receptor (CCR)3 are already directly involved in recruiting eosinophils to the lung during allergic inflammation. Once recruited, eosinophils participate in the modulation of immune response, induction of airway hyperresponsiveness and remodeling, characteristic features of asthma. Various types of promising treatments for reducing asthmatic response are related to reduction in eosinophil counts both in human and experimental models of pulmonary allergic inflammation, showing that the recruitment of these cells really plays an important role in the pathophysiology of allergic diseases such asthma.

## Introduction

Eosinophils are circulating granulocytes produced in the bone marrow along with other white blood cells and travel at relatively low levels in the bloodstream, making up 1–3% of white blood cells. These are the major cell types that can be recruited to sites of immunological or inflammatory responses (Huang et al., 2009; Isobe et al., 2012; Uhm et al., 2012). The effector function of eosinophils is related to their release of toxic granule proteins, reactive oxygen species (ROS), cytokines, and lipid mediators (Liu et al., 2006). Although eosinophils has been traditionally considered as cytotoxic effectors cells, new insights into molecular pathways allowed a better understanding of the immunomodulatory functions of these cells (Rosenberg et al., 2013). More recently, eosinophils have also been demonstrated to participate in host defense against respiratory viruses (Foster et al., 2008). With the capacity of the eosinophils to store preformed cytokines, chemokines, and growth factors available for immediate use, they play multiple roles favoring the initiation and maintenance of immune responses in inflammation, besides maintaining epithelial barrier function, affecting tissue remodeling, and bridging innate and adaptive immunity (Spencer et al., 2009; Shamri et al., 2011). The local accumulation of eosinophils is involved in the pathogenesis of allergic diseases such as asthma (Kato et al., 2006; Isobe et al., 2012; Uhm et al., 2012).

**Figure 1:**
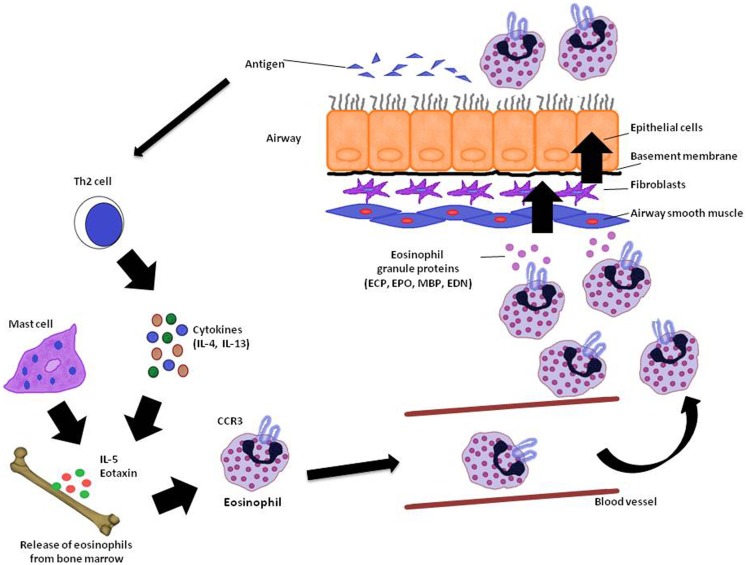
**Eosinophil recruitment in asthma**. In the reaction of the airway to antigen, Th2 cytokine producing T-cells as well as IL-5 and eotaxin stimulates the release of eosinophils from bone marrow. Elements like CC chemokine receptor (CCR3) also acting on eosinophil recruitment to the lung. When eosinophils reach the airways through the vasculature, they release granule proteins (ECP, EPO, MBP, and EDN) with cytotoxic, immunological, and remodeling-promoting properties in the lungs. ECP, eosinophil cationic protein; EPO, eosinophil peroxidase; MBP, major basic protein; EDN, eosinophil-derived neurotoxin. Adapted from Ghosh et al. (2013).

Asthma is a chronic inflammatory disease in which several inflammatory cells and mediators plays a role (Kikkawa et al., 2012). Allergic asthma is associated with eosinophilic inflammation in the airways (Lu et al., 2010). The proinflammatory mediators derived by eosinophil are major contributors to inflammation in asthma, including airway epithelial cell damage and loss, airway dysfunction of cholinergic nerve receptors, airway hyperresponsiveness, mucus hypersecretion, and airway remodeling, characterized by fibrosis and collagen deposition (Kay, 2005; Watt et al., 2005; Kanda et al., 2009; Walsh, 2010). Currently, the term “eosinophilic asthma” has been used to characterize an asthma phenotype with prevalence of eosinophils in the bronchial airways, and this phenotype can be identified by peripheral eosinophil count (Liang et al., 2012; Molfino, 2012; Spector and Tan, 2012). Figure 1 summarizes the mode of action of eosinophils and its importance to asthma.

Although eosinophils are cells studied for decades, many of the mechanisms involved in eosinophilic inflammation and infiltration in tissues are still poorly understood (Akuthota et al., 2011), mainly because eosinophils are likely to have both agonist and antagonist activities on a variety of tissue resident immune cells, such as mast cells (Lee et al., 2010).

Therefore, studies of eosinophils in human and animal models of allergic airway inflammation help us to better understand the importance of these cells in the pathogenesis of asthma.

## Molecular Events in Eosinophil Recruitment in Allergic Asthma

Molecular mechanisms are involved in complex cell-signaling pathways in allergic asthma. In this context, the activities of kinases deserves much attention (Pouliot and Olivier, 2009). An important kinase involved in the traffic and recruitment of eosinophils during allergic inflammation is the phosphatidylinositol 3-kinase (PI3K), belonging to a family of signaling molecules. These molecules regulates the adhesion, distribution, and morphologic changes of eosinophils, favoring the recruitment of these cells during lung inflammation (Kang et al., 2012). Experimental studies have been shown previously that PI3K inhibition attenuates eosinophil recruitment into airways in models of asthma (Lim et al., 2009; Park et al., 2010).

Rho-kinase is another important molecule involved in eosinophil recruitment in asthma. It has also been shown that these molecules participating in the infiltration of inflammatory cells into the airways in allergic inflammation, probably suppressing chemokine and cytokines, such as eotaxin, IL-5 and IL-13, related to the pathophysiology of asthma, subject that will be discussed further below (Taki et al., 2007; Possa et al., 2012).

Leukotrienes are another important set of molecules currently related to eosinophilic infiltration in asthma. Especially cysteinyl leukotrienes, mediators, and modulators in the pathophysiology of asthma, are related to the survival and recruitment of eosinophils to inflamed tissue (Busse and Kraft, 2005). Furthermore, leukotriene B(4) [LTB(4)] have been related to activation and recruitment of various types of inflammatory cells, among them eosinophils (Ohnishi et al., 2008). The beneficial effects in allergic inflammation and eosinophil infiltration achieved with the use of leukotrienes inhibitors will be discussed below. Anyway, it is important to emphasize here that eosinophilic recruitment into the inflamed airway in asthma is dependent of important molecular and cellular mechanisms.

## Eosinophils and Pulmonary Inflammation

Despite the controversy surrounding the true effector function of eosinophils in allergic airway inflammation, several studies have been demonstrating the importance of eosinophils in different pathways in experimental models of asthma. Modulation of immune response, induction of airway hyperresponsiveness and remodeling appear to be the main function of the recruited eosinophils in asthma (Trivedi and Lloyd, 2007).

## Immune Response

Asthma is an inflammatory disease in which multiple inflammatory mediators and modulators play function. In asthma, eosinophils are found in increased numbers in the circulation and sputum (Busse and Sedgwick, 1992). Allergen challenge in animal models of allergic inflammation is related to eosinophilia in bone marrow, blood, and lung (Lu et al., 2010). They are cells with multiple functions that interact complexly with other immune cells and their local environment (Akuthota et al., 2011).

With the allergic reaction of the airway to antigen, a cascade of processes directed by Th2 cytokine producing T-cells starts, which results in the attraction of eosinophils to the airway (Busse and Sedgwick, 1992; Wardlaw, 1999). As a consequence of exposure to the antigen, the release of eosinophils from bone marrow is mainly influenced by interleukin (IL)-5 and specific chemoattractants, such as eotaxin (Brightling, 2001). Recent investigation revealed Clara cells, important part of the “immunomodulatory barrier” of the airway epithelium, as the principal source of eotaxin in the lung (Sonar et al., 2012). It is also believed that exposure to the antigen induces trafficking of IL-5-producing T lymphocytes to the bone marrow, further promoting eosinophilopoiesis through IL-5 signaling (Gauvreau et al., 2009). IL-13 is also related to eosinophilia in asthma. This cytokine acts on IL-5 and eotaxin, which in turn selectively stimulate eosinophils. IL-5 is related to eosinophilia throughout the lung, whereas eotaxin regulates the distribution of airways eosinophils (Pope et al., 2001).

However, during episodes of allergic asthma, other elements like CC chemokine receptor (CCR)3 are already directly involved in recruiting inflammatory cells, particularly eosinophils, to the lung (Das et al., 2006). The Clara cells contribute to the infiltration of eotaxin-responsive CCR3+ immune cells and augment the allergic immune response in the lung (Sonar et al., 2012). Thus, IL-5 and IL-13 signaling for Th2 cell function are not necessarily mutually exclusive effectors mechanisms in eosinophil recruitment, but participate together with other pathways to regulate the allergic disease (Mattes and Foster, 2003).

Thus, even in the absence of IL-5, other factors including a cascade of vascular cell adhesion molecule-1, intercellular cell adhesion molecule-1, CC chemokines, and granulocyte-macrophage colony-stimulating factor (GM-CSF) are capable of maintain sufficient eosinophilic infiltration and their effector functions (Kato et al., 2006).

After attraction, locally generated IL-4 and IL-13 promote increased adhesion of the target organ vasculature to the eosinophils, and factors such as IL-5 and GM-CSF promote the local survival of eosinophils for long periods (Brightling, 2001). Even factors expressed by airway epithelial cells, including nerve growth factor and brain-derived neurotrophic factor (BDNF), promote survival of tissue eosinophils during allergic airway inflammation (Hahn et al., 2006).

Eosinophils regulate the immune response through direct effects on T-cell activities (Jacobsen et al., 2012). Both Th1 and Th2 cytokine generation by CD4(+) T-cells are also influenced by eosinophils (Liu et al., 2006; Esnault et al., 2012). In brief, eosinophils regulate the allergen-dependent Th2 pulmonary immune responses mediated by dendritic cells and T lymphocytes, as well as suppress Th1 responses (Jacobsen et al., 2011). And the eosinophils only release their granule proteins with cytotoxic, immunological, and remodeling-promoting properties when they reach the target organ, in this case the lungs, showing that these cells have basically local effects on inflammation (Malm-Erjefält et al., 2005).

When activated, eosinophils release leukotrienes (products of oxidative metabolism) and other substances such as growth factors and metalloproteinases involved in airway remodeling. The leukotrienes liberated from mast cells and eosinophils are also potent bronchoconstrictors and perpetuate the migration of eosinophils to the airways (Vignola et al., 2000; Hendeles et al., 2004; Lemanske and Busse, 2010).

Neurokinins such as Substance P (SP) and Neurokinin A (NKA) are small neuropeptides that also plays a significant role in priming eosinophils in allergic inflammation (Numao and Agrawal, 1992; Tibério et al., 1997). It has been shown that neurokinins influence eosinophil chemotaxis (Weinstock et al., 1988; Sagara et al., 1993). Furthermore, Tibério et al. (2003) demonstrated that both SP and NKA contribute to eosinophil lung recruitment in distal airways and in alveolar wall, and these findings suggest that neurokinins may contribute to the development of eosinophilic inflammation in both allergic asthma and hypersensitivity pneumonitis. These data show that modulation by neurogenic response is also very important for the recruitment of eosinophils.

Even stress may amplify the eosinophilic infiltration in both airways and lung parenchyma among animals with allergic inflammation via immune response, demonstrating that these cells probably contribute to asthmatic response related to stress (Leick et al., 2012; Marques et al., 2012). It is well known that some modulators of the stress response have different effects on eosinophil recruitment (Nittoh et al., 1998; Machida et al., 2005), but is important to note that the complexity of these interactions still needs better clarifying.

Thereafter, a series of events contribute to the arrival of eosinophils in the airways, promoting obstruction, injury, and bronchial hyperresponsiveness (Busse and Sedgwick, 1992; Wardlaw, 1999).

## Airway Hyperresponsiveness

The association between airway hyperresponsiveness and inflammation is a characteristic feature of asthma [Global Initiative for Asthma (GINA), 2011]. And airway hyperresponsiveness is a well-established consequence of eosinophil infiltration (Kay, 2005; Watt et al., 2005; Kanda et al., 2009; Kim and Lee, 2009; Walsh, 2010). There is evidence that eosinophils are involved in the bronchial hyperresponsiveness mediated by T-cell (Ohtomo et al., 2010). Iwashita et al. (2006) showed in a murine model of airway hyperresponsiveness that eosinophil chemotactic factor by T lymphocytes (ECF-L) expression was observed soon after allergen exposure but before the onset of airway inflammation, indicating that ECF-L is a selectively expressed protein in the airway hyperresponsiveness and may play a critical role in allergic inflammation.

The participation of IL-5 in stimulating eosinophils to induce airway hyperresponsiveness is clear, once depletion of eosinophils with antibody to IL-5 leads to blockage of hyperresponsiveness (Kraneveld et al., 1997; Adamko et al., 1999; Leckie et al., 2000). Furthermore, the direct effect of eosinophils in airway hyperresponsiveness has already been previously demonstrated (Gundel et al., 1991; Kanda et al., 2009). In ovalbumin (OVA)-sensitized guinea pigs (GP), the treatment with antibody to eosinophil major basic protein prevented hyperresponsiveness. It seems like eosinophil major basic protein leads to inhibition of neuronal M2 muscarinic receptor function on parasympathetic nerves in the lungs, causing airway hyperresponsiveness (Evans et al., 1997).

Assuming that total eosinophil counts reflect asthmatic activity and are useful for early detection of exacerbations, Schwartz et al. (2012) demonstrated that there is a strong association between eosinophil count in peripheral blood and airway hyperresponsiveness. Clinically, the quantity of eosinophils in the airways and sputum is directly related to the degree of airway hyperresponsiveness (Gibson et al., 2000; Obase et al., 2001).

## Remodeling

Airway remodeling is the cellular and structural changes in the airways, mainly resulting from repair processes in response to persistent inflammation, and that contribute to irreversibility of lung functions observed in asthma patients, including airway dysfunction and clinical symptoms observed in allergic asthma (Vignola et al., 2000, 2003; Phipps et al., 2004; Fattouh and Jordana, 2008). Among structural changes can be noted subepithelial fibrosis, smooth muscle hypertrophy/hyperplasia, epithelial cell mucus metaplasia, and increased angiogenesis (Aceves and Broide, 2008).

Eosinophils seem to contribute to airway remodeling in several ways, including through release of eosinophil-derived mediators such as transforming growth factor (TGF)-β, secretion of cationic proteins, and cytokines, as well as through interactions with mast cell and epithelial cells. Many of these factors can directly activate epithelium and mesenchymal cells, deeply related to the development of airway remodeling (Kariyawasam and Robinson, 2007; Aceves and Broide, 2008; Venge, 2010). Eosinophil-derived cytokines are in the modulation of Th2 responses that trigger macrophage production of TGF-β_1_, which serves as a stimulus for extracellular matrix production (Fanta et al., 1999; Holgate, 2001).

In addition, recent data demonstrated that eosinophils can also contribute to airway remodeling during asthma by enhancing airway smooth muscle (ASM) cell proliferation. Halwani et al. (2013) verified that preventing eosinophil contact with ASM cells using specific antibodies or blocking cysteinyl leukotrienes derived from eosinophils was associated with inhibition of ASM proliferation. Moreover, ASM-synthesized cytokines seem to direct the eosinophil differentiation and maturation from progenitor cells, which can promote perpetuation of eosinophilic inflammation and consequently the tissue remodeling in asthma (Fanat et al., 2009).

## Eosinophils in Lung Parenchyma

The involvement of the lung parenchyma in the pathophysiology of asthma is already well-established. Recently, it has been recognized that these distal lung alterations can corroborate global pulmonary alterations, enhancing asthma symptoms (Dolhnikoff et al., 1999; Rocco et al., 2001; Mauad et al., 2004; Xisto et al., 2005; Lancas et al., 2006).

In this context, several authors have shown that eosinophilic inflammation were present in the peripheral lung parenchyma of asthmatic patients and in experimental animal models of chronic pulmonary inflammation (Mauad et al., 2004; Xisto et al., 2005; Lancas et al., 2006; Angeli et al., 2008; Araujo et al., 2008; Nakashima et al., 2008; Starling et al., 2009). Thus, it is important to note that not only the airways receive influence of eosinophil recruitment in asthma, but that it also occurs in the lung parenchyma.

## Relationship between Experimental Treatments for Chronic Pulmonary Allergic Inflammation and Eosinophils

Various types of promising treatments for reducing asthmatic response are related to reduction in eosinophil counts both in human and experimental models of pulmonary allergic inflammation, showing that these cells really play an important role in the pathophysiology of asthma.

Guinea pigs chronically exposed to OVA present an inflammatory response predominantly eosinophilic, showing a significant increase in eosinophil amount in airways, pulmonary parenchyma, and bronchoalveolar lavage (BAL), constituting an interesting experimental model to evaluate the participation of eosinophils in allergic inflammation (Tibério et al., 1997; Leick-Maldonado et al., 2004; Prado et al., 2005a,b; Lancas et al., 2006; Angeli et al., 2008; Nakashima et al., 2008; Ruiz-Schütz et al., 2009; Leick et al., 2012; Marques et al., 2012; Possa et al., 2012).

### Anti-leukotrienes

Cysteinyl leukotrienes are proinflammatory mediators with many pulmonary actions, including human ASM contraction, chemotaxis, mucous secretion, smooth muscle proliferation, and increase in vascular permeability (Drazen, 1998; Drazen et al., 1999; Holgate and Sampson, 2000; O’Byrne, 2000). *In vivo* and *in vitro* influences of leukotrienes in chemotaxis of eosinophils have been previously shown. The treatment with montelukast, an anti-leukotriene drug, was able to reduce eosinophils, with effects similar to dexamethasone treatment, in an experimental model of pulmonary allergic inflammation in GP. The findings were correlated to attenuation of airway response (Leick-Maldonado et al., 2004).

Blain and Sirois (2000) showed in sensitized mice that there was a dose-dependent reduction in eosinophils in BAL by both dexamethasone and cysteinyl leukotriene-receptor antagonist. Muraki et al. (2011) also used cysteinyl leukotrienes receptor antagonists in OVA-sensitized GP and have found significant suppression of eosinophil proliferation into BAL fluid and airways walls (Muraki et al., 2011). Foster and Chan (1991) showed, in sensitized GP, that the increase in eosinophil influx into airway submucosa was attenuated by using a leukotriene-receptor antagonist. Henderson et al. (2002) observed that montelukast treatment resulted in a reduction of eosinophil infiltration in lung interstitium of mice with chronic inflammation induced by OVA exposure.

Factors driving eosinophil influx induced by leukotrienes may include IL-5 altered eosinophilopoiesis and release from the bone marrow, reduced priming of eosinophils, altering the expression of adhesion molecules, and reduced eosinophil apoptosis (Busse, 2001).

An *in vitro* study with montelukast showed that this antagonist has inhibitory effects on epithelial cell cytokine secretion, including secretion of IL-6 and IL-8, as well as on eosinophil survival, suggesting the mechanisms by which leukotrienes exert their functions on eosinophils in inflammation (Mullol et al., 2010).

### Nitric oxide inhibition

It has already been demonstrated acute Nitric oxide (NO) inhibition, but not chronic treatment, by *N*^ω^-nitro-l-arginine methyl ester (l-NAME) is associated with reduction of eosinophils in the airway wall and lung parenchyma of OVA-exposed GP with chronic pulmonary allergic inflammation, showing that NO plays an important role in inflammatory cell recruitment (Prado et al., 2005a,b; Angeli et al., 2008). The acute effects of NO inhibitors on inflammatory cell recruitment have also been observed by other authors (Feder et al., 1997; Schuiling et al., 1998).

Furthermore, it has been shown that l-NAME treatment reduces the number of eosinophils positive for both neuronal nitric oxide synthase (nNOS) and inducible nitric oxide synthase (iNOS), while the treatment with 1400W, a highly selective iNOS inhibitor, reduce only the iNOS-positive eosinophils, without modifying the number of cells positive for nNOS (Prado et al., 2006). Starling et al. (2009) also found that iNOS-specific inhibition with 1400W reduces the eosinophil density in alveolar septa of allergen-sensitized animals. These results confirm not only the effectiveness of both treatments in exhaled NO reduction, but also that NO production is very important to eosinophilic recruitment.

Although there are several studies showing a role of NO in inflammatory cell recruitment, no effects in eosinophil recruitment are still a matter of controversy. Some authors showed that acute treatment with non-selective inhibitors of NO reduced allergen-induced eosinophilia (Feder et al., 1997; Iijima et al., 2001). However, Eynott et al. (2002) demonstrated that specific inhibition of iNOS reduced only neutrophils. Blease et al. (2000) showed that single l-NAME dose increased peribronchial and BAL fluid eosinophils in a murine model of fungal asthma. Ferreira et al. (1998) demonstrated that chronic l-NAME treatment reduced eosinophils in a model of acute inflammation. A recent study showed that NO induces eosinophil apoptosis in a mechanism mediated via ROS, c-jun N-terminal kinase (JNK), and later mitochondrial permeability transition (mPT) (Ilmarinen-Salo et al., 2012).

These conflicting data between results may be related to the fact that different protocols of antigen sensitization, antigen challenge, type of inhibitors used, and different species have been used. Moreover, the concentration, flux and source of NO influencing eosinophilopoiesis, eosinophilic recruitment, and apoptosis, with either anti- or pro-apoptotic properties may influence the obtained results (Taylor et al., 2003).

### Oral tolerance

Oral tolerance is associated with reduction of eosinophil recruitment into distal airways and lung parenchyma, response that is associated with attenuation of airways and lung tissue hyperresponsiveness, as well with reduction in collagen and elastic fiber deposition (Nakashima et al., 2008; Ruiz-Schütz et al., 2009).

Some authors also investigated the eosinophilic response around the airways and speculated that the development of the tolerance process was associated with the disappearance of the Th2 lymphocyte population (Russo et al., 1998, 2001; Chung et al., 2002; Keller et al., 2006). Vaickus et al. (2010) compared the allergic pulmonary inflammation of allergen-sensitized mice submitted to oral tolerance treatment with different types of complex mixture of insect components, and verified that oral tolerance was related to reduction in eosinophil numbers in the BAL fluid and eosinophil specific peroxidase activity in the lung homogenate, demonstrating that oral tolerization is associated with reduction in pulmonary eosinophilia. The decrease in eosinophilopoiesis with oral tolerance demonstrates the idea of the importance of controlling the eosinophilic inflammatory response by immune response modulation (Ruiz-Schütz et al., 2009).

### Rho-kinase inhibition

Rho-kinase is an effector protein of the Rho/Rho-kinase pathway that is associated with Ca^2+^ sensitization to promote smooth muscle contraction (Yoshii et al., 1999). Despite Rho-kinase is mainly associated with airway hyperresponsiveness (Hashimoto et al., 2002; Schaafsma et al., 2004, 2008), the infiltration of inflammatory cells into the airways (Taki et al., 2007; Schaafsma et al., 2008) is other important function of this effector protein.

It has been recently demonstrated that the specific Rho-kinase inhibition is associated with reduction in eosinophil recruitment into airways in GP with chronic pulmonary allergic inflammation (Possa et al., 2012).

Several studies have suggested that the RhoA/ROCK system plays a role in eosinophil recruitment and Th1 and Th2 cytokine secretion (Aihara et al., 2003, 2004; Henry et al., 2005). In this regard, Henry et al. (2005) demonstrated that pretreatment with Y-27632 reduced the number of eosinophils recovered from the BAL fluid of OVA-sensitized mice. Taki et al. (2007) demonstrated that another Rho-kinase inhibitor, fasudil, reduced the numbers of eosinophils in BAL fluid, airways and blood vessels. This Rho-kinase inhibitor also diminished the augmented levels of IL-5, IL-13, and eotaxin in BAL fluid, demonstrating that Rho-kinase pathway influences modulators of eosinophilic recruitment.

Zhu et al. (2011) showed that at least two Rho-kinase isoforms, ROCK1 and ROCK2, are associated with eosinophilic recruitment in a model of OVA-challenged mice. Such results lead us to think that both treatment with inhibitors of Rho-kinase as with other drugs for suppressing eosinophilic inflammation and consequently its deleterious effects, would be very beneficial in the treatment of asthma.

Table 1 summarizes the several possible ways of eosinophil recruitment in asthma, with some important references.

**Table 1:** **Mechanisms of eosinophil recruitment in asthma**.

Mechanisms related to eosinophil infiltration	Reference
IL-5, Il-13, and eotaxin	Brightling (2001), Pope et al. (2001), Gauvreau et al. (2009)
CCR3	Mattes and Foster (2003), Das et al. (2006), Sonar et al. (2012)
Neurokinins	Weinstock et al. (1988), Numao and Agrawal (1992), Sagara et al. (1993), Tibério et al. (1997)
Stress	Nittoh et al. (1998), Machida et al. (2005), Leick et al. (2012), Marques et al. (2012)
Cysteinyl leukotrienes	Foster and Chan (1991), Blain and Sirois (2000), Leick-Maldonado et al. (2004), Muraki et al. (2011)
Nitric oxide	Feder et al. (1997), Ferreira et al. (1998), Iijima et al. (2001), Prado et al. (2006), Starling et al. (2009), Ilmarinen-Salo et al. (2012)
Oral tolerance	Russo et al. (1998, 2001), Chung et al. (2002), Keller et al. (2006), Nakashima et al. (2008), Ruiz-Schütz et al. (2009), Vaickus et al. (2010)
Rho-kinase	Aihara et al. (2003, 2004), Henry et al. (2005), Taki et al. (2007), Zhu et al. (2011), Possa et al. (2012)

There are still controversies about if eliminating eosinophils is a risk-free therapeutic strategy. Since eosinophils contribute to defense against respiratory viruses, the elimination of these cells may potentially increase the risk for viral infections, which may predispose to the development of acute exacerbations of asthma, an outcome that would have significant clinical implications (Foster et al., 2008).

## Conclusion

Increasingly consistent evidence suggests that eosinophils participate in a wide variety of functions in allergic lung inflammation. In this context, it is important to consider this as a potential target cell for the treatment of asthma. However, given the importance of eosinophils in pathogenesis of asthma but also in lung defense mechanisms, one must consider that the best way to treat asthma should include not its complete elimination, but the partial control of eosinophilic response.

## Conflict of Interest Statement

The authors declare that the research was conducted in the absence of any commercial or financial relationships that could be construed as a potential conflict of interest.

## References

[B1] Aceves S. S. Broide D. H. (2008). Airway fibrosis and angiogenesis due to eosinophil trafficking in chronic asthma. Curr. Mol. Med. 8, 350–358 10.2174/156652408785161023 18691061

[B2] Adamko D. J. Yost B. L. Gleich G. J. Fryer A. D. Jacoby D. B. (1999). Ovalbumin sensitization changes the inflammatory response to subsequent parainfluenza infection. Eosinophils mediate airway hyperresponsiveness, m(2) muscarinic receptor dysfunction, and antiviral effects. J. Exp. Med. 90, 1465–1478 10.1084/jem.190.10.1465 10562321 PMC2195693

[B3] Aihara M. Dobashi K. Iizuka K. Nakazawa T. Mori M. (2003). Comparison of effects of Y-27632 and Isoprotenerol on release of cytolines from human peripheral T cells. Int. Immunopharmacol. 3, 1619–1625 10.1016/S1567-5769(03)00184-X 14555287

[B4] Aihara M. Dobashi K. Iizuka K. Nakazawa T. Mori M. (2004). Effect of Y-27632 on release of cytokines from peripheral T cells in asthmatic patients and normal subjects. Int. Immunopharmacol. 4, 557–561 10.1016/j.intimp.2003.12.014 15099533

[B5] Akuthota P. Xenakis J. J. Weller P. F. (2011). Eosinophils: offenders or general bystanders in allergic airway disease and pulmonary immunity? J. Innate Immun. 3, 113–119 10.1159/000323433 21228563 PMC3072201

[B6] Angeli P. Prado C. M. Xisto D. G. Silva P. L. Pássaro C. P. Nakazato H. D. (2008). Effects of chronic L-NAME treatment lung tissue responses induced by chronic pulmonary inflammation mechanics, eosinophilic and extracellular matrix. Am. J. Physiol. Lung Cell. Mol. Physiol. 294, L1197–L1205 10.1152/ajplung.00199.2007 18359886

[B7] Araujo B. B. Dolhnikoff M. Silva L. F. Elliot J. Lindeman J. H. Ferreira D. S. (2008). Extracellular matrix components and regulators in the airway smooth muscle in asthma. Eur. Respir. J. 32, 61–69 10.1183/09031936.00147807 18321931

[B8] Blain J. F. Sirois P. (2000). Involvement of LTD4 in allergic pulmonary inflammation in mice: modulation by cysLT1 antagonist MK-571. Prostaglandins Leukot. Essent. Fatty Acids 62, 361–368 10.1054/plef.2000.0167 10913229

[B9] Blease K. Kunkel S. L. Hogaboam C. M. (2000). Acute inhibition of nitric oxide exacerbates airway hyperresponsiveness, eosinophilia and C-C chemokine generation in a murine model of fungal asthma. Inflamm. Res. 49, 297–304 10.1007/PL00000210 10939620

[B10] Brightling C. E. (2001). Eosinophils, bronchitis and asthma: pathogenesis of cough and airflow obstruction. Pulm. Pharmacol. Ther. 24, 324–327 10.1016/j.pupt.2010.11.001 21074631

[B11] Busse W. Kraft M. (2005). Cysteinyl leukotrienes in allergic inflammation: strategic target for therapy. Chest 127, 1312–1326 10.1378/chest.127.4.1312 15821210

[B12] Busse W. W. (2001). Does leukotriene modulation of eosinophil function explain the therapeutic effectiveness of receptor antagonists in some patients with asthma? Clin. Exp. Allergy 31, 806–807 10.1046/j.1365-2222.2001.01144.x 11422142

[B13] Busse W. W. Sedgwick J. B. (1992). Eosinophils in asthma. Ann. Allergy 68, 286–290 1546825

[B14] Chung Y. Cho J. Chang Y. S. Cho S. H. Kang C. Y. (2002). Preventive and therapeutic effects of oral tolerance in a murine model of asthma. Immunobiology 206, 408–423 10.1078/0171-2985-00190 12437071

[B15] Das A. M. Vaddi K. G. Solomon K. A. Krauthauser C. Jiang X. McIntyre K. W. (2006). Selective inhibition of eosinophil influx into the lung by small molecule CC chemokine receptor 3 antagonists in mouse models of allergic inflammation. J. Pharmacol. Exp. Ther. 318, 411–417 10.1124/jpet.105.099812 16614169

[B16] Dolhnikoff M. Mauad T. Ludwig M. S. (1999). Extracellular matrix and oscillatory mechanics of rat lung parenchyma in bleomycin-induced fibrosis. Am. J. Respir. Crit. Care Med. 160, 1750–1757 10.1164/ajrccm.160.5.9812040 10556151

[B17] Drazen J. M. (1998). Leukotrienes as mediators of airway obstruction. Am. J. Respir. Crit. Care Med. 158, S193–S200 10.1164/ajrccm.158.supplement_2.13tac180 9817745

[B18] Drazen J. M. Israel E. O’Byrne P. M. (1999). Treatment of asthma with drugs modifying the leukotriene pathway. N. Engl. J. Med. 340, 197–206 10.1056/NEJM199901213400306 9895400

[B19] Esnault S. Kelly E. A. Nettenstrom L. M. Cook E. B. Seroogy C. M. Jarjour N. N. (2012). Human eosinophils release IL-1ß and increase expression of IL-17A in activated CD4(+) T lymphocytes. Clin. Exp. Allergy 42, 1756–1764 10.1111/j.1365-2222.2012.04060.x 23181791 PMC3612975

[B20] Evans C. M. Fryer A. D. Jacoby D. B. Gleich G. J. Costello R. W. (1997). Pretreatment with antibody to eosinophil major basic protein prevents hyperresponsiveness by protecting neuronal M2 muscarinic receptors in antigen-challenged guinea pigs. J. Clin. Invest. 100, 2254–2262 10.1172/JCI119763 9410903 PMC508421

[B21] Eynott P. R. Groneberg D. A. Caramori G. Adcock I. M. Donnely L. E. Kharitonov S. (2002). Role of nitric oxide in allergic inflammation and bronchial hyperresponsiveness. Eur. J. Pharmacol. 452, 123–133 10.1016/S0014-2999(02)02237-9 12323393

[B22] Fanat A. I. Thomson J. V. Radford K. Nair P. Sehmi R. (2009). Human airway smooth muscle promotes eosinophil differentiation. Clin. Exp. Allergy 39, 1009–1017 10.1111/j.1365-2222.2009.03246.x 19438586

[B23] Fanta C. Bohle B. Hirt W. Siemann U. Horak F. Kraft D. (1999). Systemic immunological changes induced by administration of grass pollen allergens via the oral mucosa during sublingual immunotherapy. Int. Arch. Allergy Immunol. 120, 218–224 10.1159/000024270 10592467

[B24] Fattouh R. Jordana M. (2008). TGF-beta, eosinophils and IL-13 in allergic airway remodeling: a critical appraisal with therapeutic considerations. Inflamm. Allergy Drug Targets 7, 224–236 10.2174/187152808786848388 19075788

[B25] Feder L. S. Stelts D. Chapman R. W. Manfra D. Crawley Y. Jones H. (1997). Role of nitric oxide on eosinophilic lung inflammation in allergic mice. Am. J. Respir. Cell. Mol. Biol. 17, 436–442 10.1165/ajrcmb.17.4.2845 9376118

[B26] Ferreira H. H. A. Bevilacqua E. Gagioti S. M. De Luca I. M. S. Zanardo R. C. O. Teixeira C. E. (1998). Nitric oxide modulates eosinophil infiltration in antigen-induced airway inflammation in rats. Eur. J. Pharmacol. 358, 253–258 10.1016/S0014-2999(98)00575-5 9822892

[B27] Foster A. Chan C. C. (1991). Peptide leukotriene involvement in pulmonary eosinophil migration upon antigen challenge in the actively sensitized guinea pig. Int. Arch. Allergy Appl. Immunol. 96, 279–284 10.1159/000235508 1666630

[B28] Foster P. S. Rosenberg H. F. Asquith K. L. Kumar R. K. (2008). Targeting eosinophils in asthma. Curr. Mol. Med. 8, 585–590 10.2174/156652408785748013 18781965 PMC3727917

[B29] Gauvreau G. M. Ellis A. K. Denburg J. A. (2009). Haemopoietic processes in allergic disease: eosinophil/basophil development. Clin. Exp. Allergy 39, 1297–1306 10.1111/j.1365-2222.2009.03325.x 19622087

[B30] Ghosh S. Hoselton S. A. Dorsam G. P. Schuh J. M. (2013). Eosinophils in fungus-associated allergic pulmonary disease. Front. Pharmacol. 4:8 10.3389/fphar.2013.00008 23378838 PMC3561640

[B31] Gibson P. G. Saltos N. Borgas T. (2000). Airway mast cells and eosinophils correlate with clinical severity and airway hyperresponsiveness in corticosteroid-treated asthma. J. Allergy Clin. Immunol. 105, 752–759 10.1067/mai.2000.105319 10756226

[B32] Global Initiative for Asthma (GINA) (2011). Global Strategy for Asthma Management and Prevention. Available at: http://www.ginasthma.org

[B33] Gundel R. H. Letts L. G. Gleich G. J. (1991). Human eosinophil major basic protein induces airway constriction and airway hyperresponsiveness in primates. J. Clin. Invest. 87, 1470–1473 10.1172/JCI115155 2010556 PMC295201

[B34] Hahn C. Islamian A. P. Renz H. Nockher W. A. (2006). Airway epithelial cells produce neurotrophins and promote the survival of eosinophils during allergic airway inflammation. J. Allergy Clin. Immunol. 117, 787–794 10.1016/j.jaci.2005.12.1339 16630935

[B35] Halwani R. Vazquez-Tello A. Sumi Y. Pureza M. A. Bahammam A. Al-Jahdali H. (2013). Eosinophils induce airway smooth muscle cell proliferation. J. Clin. Immunol. 33, 595–604 10.1007/s10875-012-9836-3 23180361

[B36] Hashimoto T. Nakano Y. Yamashita M. Fang Y. Ohata H. Momose K. (2002). Role of Rho-associated protein kinase and histamine in lysophosphatidic acid induced airway hyperresponsiveness in guinea pigs. Jpn. J. Pharmacol. 88, 256–261 10.1254/jjp.88.256 11949879

[B37] Hendeles L. Asmus M. Chresrown S. (2004). Evaluation of cytokine modulators for asthma. Paediatr. Respir. Rev. 25, 107–112 10.1016/S1526-0542(04)90020-6 14980253

[B38] Henderson W. R. Jr. Tang L. O. Chu S. J. Tsao S. M. Chiang G. K. Jones F. (2002). A role for cysteinyl leukotrienes in airway remodeling in a mouse asthma model. Am. J. Respir. Crit. Care Med. 165, 108–116 10.1164/ajrccm.165.1.2105051 11779739

[B39] Henry P. J. Mann T. S. Goldie R. G. (2005). A Rho kinase inhibitor, Y-27632 inhibits pulmonary eosinophilia, bronchoconstriction and airways hyperresponsiveness in allergic mice. Pulm. Pharmacol. Ther. 18, 67–74 10.1016/j.pupt.2004.10.002 15607129

[B40] Holgate S. (2001). Mechanisms of allergy and adult asthma. Curr. Opin. Allergy Clin. Immunol. 1, 47–50 10.1097/00130832-200102000-00009 11964669

[B41] Holgate S. T. Sampson A. P. (2000). Antileukotriene therapy: future directions. Am. J. Respir. Crit. Care Med. 61, S147–153 10.1164/ajrccm.161.supplement_1.ltta-29 10673245

[B42] Huang F. Y. Wang C. C. Zhou S. L. Huang Y. H. Wang H. Chen F. (2009). Antisense interleukin-5 reduces eosinophil infiltration and hyperresponsiveness in an allergic asthma model. Asian Pac. J. Allergy Immunol. 27, 35–41 19548628

[B43] Iijima H. Duguet A. Eum S. Y. Hamid Q. Eidelman D. H. (2001). Nitric oxide and protein nitration are eosinophil dependent in allergen-challenged mice. Am. J. Respir. Crit. Care Med. 163, 1233–1240 10.1164/ajrccm.163.5.2003145 11316664

[B44] Ilmarinen-Salo P. Moilanen E. Kinnula V. L. Kankaanranta H. (2012). Nitric oxide-induced eosinophil apoptosis is dependent on mitochondrial permeability transition (mPT), JNK and oxidative stress: apoptosis is preceded but not mediated by early mPT-dependent JNK activation. Respir. Res. 13, 73 10.1186/1465-9921-13-73 22920281 PMC3495716

[B45] Isobe Y. Kato T. Arita M. (2012). Emerging roles of eosinophils and eosinophil-derived lipid mediators in the resolution of inflammation. Front. Immunol. 3:270 10.3389/fimmu.2012.00270 22973272 PMC3428698

[B46] Iwashita H. Morita S. Sagiya Y. Nakanishi A. (2006). Role of eosinophil chemotactic factor by T lymphocytes on airway hyperresponsiveness in a murine model of allergic asthma. Am. J. Respir. Cell. Mol. Biol. 35, 103–109 10.1165/rcmb.2005-0134OC 16528013

[B47] Jacobsen A. Helmers R. A. Lee J. J. Lee N. A. (2012). The expanding role(s) of eosinophils in health and disease. Blood 120, 3882–3890 10.1182/blood-2012-06-330845 22936660 PMC3496950

[B48] Jacobsen E. A. Zellner K. R. Colbert D. Lee N. A. Lee J. J. (2011). Eosinophils regulate dendritic cells and Th2 pulmonary immune responses following allergen provocation. J. Immunol. 187, 6059–6068 10.4049/jimmunol.1102299 22048766 PMC3375323

[B49] Kanda A. Driss V. Hornez N. Abdallah M. Roumier T. Abboud G. (2009). Eosinophil-derived IFN-gamma induces airway hyperresponsiveness and lung inflammation in the absence of lymphocytes. J. Allergy Clin. Immunol. 124, 573–582 10.1016/j.jaci.2009.04.031 19539982

[B50] Kang B. N. Ha S. G. Ge X. N. Reza Hosseinkhani M. Bahaie N. S. Greenberg Y. (2012). The p110δ subunit of PI3K regulates bone marrow-derived eosinophil trafficking and airway eosinophilia in allergen-challenge mice. Am. J. Physiol. Lung Cell. Mol. Physiol. 302, L1179–1191 10.1152/ajplung.00005.2012 22427531 PMC3379039

[B51] Kariyawasam H. H. Robinson D. S. (2007). The role of eosinophils in airway tissue remodelling in asthma. Curr. Opin. Immunol. 19, 681–686 10.1016/j.coi.2007.07.021 17949963

[B52] Kato M. Suzuki M. Hayashi Y. Kimura H. (2006). Role of eosinophils and their clinical significance in allergic inflammation. Expert Rev. Clin. Immunol. 2, 121–133 10.1586/1744666X.2.1.121 20477093

[B53] Kay A. B. (2005). The role of eosinophils in the pathogenesis of asthma. Trends Mol. Med. 11, 148–152 10.1016/j.molmed.2005.02.002 15823751

[B54] Keller A. C. Mucida D. Gomes E. Faquim-Mauro E. Faria A. M. Rodriguez D. (2006). Hierarchical suppression of asthma-like responses by mucosal tolerance. J. Allergy Clin. Immunol. 117, 283–290 10.1016/j.jaci.2005.12.1171 16461128

[B55] Kikkawa Y. Sugiyama K. Obara K. Hirata H. Fukushima Y. Toda M. (2012). Interferon-alpha inhibits airway eosinophila and hyperresponsiveness in an animal asthma model. Asia Pac. Allergy 2, 256–263 10.5415/apallergy.2012.2.4.256 23130331 PMC3486970

[B56] Kim S. H. Lee Y. C. (2009). Piperine inhibits eosinophil infiltration and airway hyperresponsiveness by suppressing T cell activity and Th2 cytokine production in the ovalbumin-induced asthma model. J. Pharm. Pharmacol. 61, 353–359 10.1211/jpp.61.03.0010 19222908

[B57] Kraneveld A. D. van Ark I. Van Der Linde H. J. Fattah D. Nijkamp F. P. Van Oosterhout A. J. (1997). Antibody to very late activation antigen 4 prevents interleukin-5-induced airway hyperresponsiveness and eosinophil infiltration in the airways of guinea pigs. J. Allergy Clin. Immunol. 100, 242–250 10.1016/S0091-6749(97)70231-8 9275147

[B58] Lancas T. Kasahara D. I. Prado C. M. Tiberio I. F. Martins M. A. Dolhnikoff M. (2006). Comparison of early and late responses to antigen of sensitized guinea pig parenchymal lung strips. J. Appl. Physiol. 100, 1610–1616 10.1152/japplphysiol.00828.2005 16410372

[B59] Leckie M. J. ten Brinke A. Khan J. Diamant Z. O’Connor B. J. Walls C. M. (2000). Effects of an interleukin-5 blocking monoclonal antibody on eosinophils, airway hyperresponsiveness, and the late asthmatic response. Lancet 356, 2144–2148 10.1016/S0140-6736(00)03496-6 11191542

[B60] Lee J. J. Jacobsen E. A. McGarry M. P. Schleimer R. P. Lee N. A. (2010). Eosinophils in health and disease: the LIAR hypothesis. Clin. Exp. Allergy 40, 563–575 10.1111/j.1365-2222.2010.03484.x 20447076 PMC2951476

[B61] Leick E. A. Reis F. G. Honorio-Neves F. A. Almeida-Reis R. Prado C. M. Martins M. A. (2012). Effects of repeated stress on distal airway inflammation, remodeling and mechanics in an animal model of chronic airway inflammation. Neuroimmunomodulation 19, 1–9 10.1159/000324686 22067616

[B62] Leick-Maldonado E. A. Kay F. U. Leonhardt M. C. Kasahara D. I. Prado C. M. Fernandes F. T. (2004). Comparison of glucocorticoid and cysteinyl leukotriene receptor antagonist treatments in an experimental model of chronic airway inflammation in guinea-pigs. Clin. Exp. Allergy 34, 145–152 10.1111/j.1365-2222.2004.01854.x 14720275

[B63] Lemanske R. F. Busse W. W. (2010). Asthma: clinical expression and molecular mechanisms. J Allergy Clin Immunol. 125, 95–102 10.1016/j.jaci.2009.10.047 PMC2853245 20176271

[B64] Liang Z. Zhao H. Lv Y. Li R. Dong H. Liu L. (2012). Moderate accuracy of peripheral eosinophil count for predicting eosinophilic phenotype in steroid-naïve non-atopic adult asthmatics. Intern. Med. 51, 717–722 10.2169/internalmedicine.51.6834 22466826

[B65] Lim D. H. Cho J. Y. Sopng D. J. Lee S. Y. Miller M. Broide D. H. (2009). PI3K gamma-defficient mice have reduced levels of allergen-induced eosinophilic inflammation and airway remodeling. Am. J. Physiol. Lung Cell. Mol. Physiol. 296, L210–219 10.1152/ajplung.90275.2008 19028980 PMC2643991

[B66] Liu L. Y. Mathur S. K. Sedgwick J. B. Jarjour N. N. Busse W. W. Kelly E. (2006). A. Human airway and peripheral blood eosinophils enhance Th1 and Th2 cytokine secretion. Allergy 61, 589–597 10.1111/j.1398-9995.2006.01060.x 16629789

[B67] Lu Y. Sjöstrand M. Malmhäll C. Rådinger M. Jeurink P. Lötvall J. (2010). New production of eosinophils and the corresponding TH1/TH2 balance in the lungs after allergen exposure in BALB/c and C57BL/6 mice. Scand. J. Immunol. 71, 176–185 10.1111/j.1365-3083.2009.02364.x 20415783

[B68] Machida K. Inoue H. Matsumoto K. Tsuda M. Fukuyama S. Koto H. (2005). Activation of PI3KAkt pathway mediates antiapoptotic effects of beta-adrenergic agonist in airway eosinophils. Am. J. Physiol. Lung Cell. Mol. Physiol. 288, L860–L867 10.1152/ajplung.00131.2004 15618457

[B69] Malm-Erjefält M. Greiff L. Ankerst J. Andersson M. Wallengren J. Cardell L. O. (2005). Circulating eosinophils in asthma, allergic rhinitis, and atopic dermatitis lack morphological signs of degranulation. Clin. Exp. Allergy 35, 1334–1340 10.1111/j.1365-2222.2005.02335.x 16238793

[B70] Marques R. H. Reis F. G. Starling C. M. Cabido C. de Almeida-Reis R. Dohlnikoff M. (2012). Inducible nitric oxide synthase inhibition attenuates physical stress-induced lung hyperresponsiveness and oxidative stress in animals with lung inflammation. Neuroimmunomodulation 19, 158–170 10.1159/000331264 22262048

[B71] Mattes J. Foster P. S. (2003). Regulation of eosinophil migration and Th2 cell function by IL-5 and eotaxin. Curr. Drug Targets Inflamm. Allergy 2, 169–174 10.2174/1568010033484214 14561170

[B72] Mauad T. Silva L. F. Santos M. A. Grinberg L. Bernardi F. D. Martins M. A. (2004). Abnormal alveolar attachments with decreased elastic fiber content in distal lung in fatal asthma. Am. J. Respir. Crit. Care Med. 170, 857–862 10.1164/rccm.200403-305OC 15151920

[B73] Molfino N. A. (2012). Targeting of eosinophils in asthma. Expert Opin. Biol. Ther. 12, 807–809 10.1517/14712598.2012.674938 22500988

[B74] Mullol J. Callejas F. B. Méndez-Arancibia E. Fuentes M. Alobid I. Martínez-Antón A. (2010). Montelukast reduces eosinophilic inflammation by inhibiting both epithelial cell cytokine secretion (GM-CSF, IL-6, IL-8) and eosinophil survival. J. Biol. Regul. Homeost. Agents 24, 403–411 21122279

[B75] Muraki M. Imbe S. Santo H. Sato R. Sano H. Iwanaga T. (2011). Effects of a cysteinyl leukotriene dual 1/2 receptor antagonist on antigen-induced airway hypersensitivity and airway inflammation in a guinea pig asthma model. Int. Arch. Allergy Immunol. 155, 90–95 10.1159/000327439 21646802

[B76] Nakashima A. S. Prado C. M. Lancas T. Ruiz V. C. Kasahara D. I. Leick-Maldonado E. A. (2008). Oral tolerance attenuates changes in in vitro lung tissue mechanics and extracellular matrix remodeling induced by chronic allergic inflammation in guinea pigs. J. Appl. Physiol. 104, 1778–1785 10.1152/japplphysiol.00830.2007 18388250

[B77] Nittoh T. Fujimori H. Kozumi Y. Ishihara K. Mue S. Ohuchi K. (1998). Effects of glucocorticoids on apoptosis of infiltrated eosinophils and neutrophils in rats. Eur. J. Pharmacol. 354, 73–81 10.1016/S0014-2999(98)00426-9 9726633

[B78] Numao T. Agrawal D. K. (1992). Neuropeptides modulate human eosinophil chemotaxis. J. Immunol. 149, 3309–3315 1385521

[B79] Obase Y. Shimoda T. Mitsuta K. Matsuo N. Matsuse H. Kohno S. (2001). Correlation between airway hyperresponsiveness and airway inflammation in a young adult population: eosinophil, ECP, and cytokine levels in induced sputum. Ann. Allergy Asthma Immunol. 86, 304–310 10.1016/S1081-1206(10)63303-0 11289329

[B80] O’Byrne P. M. (2000). Leukotriene bronchoconstriction induced by allergen and exercises. Am. J. Respir. Crit. Care Med. 161, S68–72 10.1164/ajrccm.161.supplement_2.a1q4-8 10673230

[B81] Ohnishi H. Miyahara N. Gelfand E. W. (2008). The role of leukotrien B(4) in allergic diseases. Allergol. Int. 57, 291–298 10.2332/allergolint.08-RAI-0019 18797182

[B82] Ohtomo T. Kaminuma O. Yamada J. Kitamura N. Abe A. Kobayashi N. (2010). Eosinophils are required for the induction of bronchial hyperresponsiveness in a Th transfer model of BALB/c background. Int. Arch. Allergy Immunol. 152, 79–82 10.1159/000312130 20523068

[B83] Park S. J. Lee K. S. Kim S. R. Min K. H. Moon H. Lee M. H. (2010). Phosphoinositide 3-kinase δ inhibitor suppresses interleukin-17 expression in a murine asthma model. Eur. Respir. J. 36, 1448–1459 10.1183/09031936.00106609 20351038

[B84] Phipps S. Benyahia F. Ou T. T. Barkans J. Robinson D. S. Kay A. B. (2004). Acute allergen-induced airway remodeling in atopic asthma. Am. J. Respir. Cell Mol. Biol. 31, 626–632 10.1165/rcmb.2004-0193OC 15333330

[B85] Pope S. M. Brandt E. B. Mishra A. Hogan S. P. Zimmermann N. Matthaei K. I. (2001). IL-13 induces eosinophil recruitment into the lung by an IL-5- and eotaxin-dependent mechanism. J. Allergy Clin. Immunol. 108, 594–601 10.1067/mai.2001.118600 11590387

[B86] Possa S. S. Charafeddine H. T. Righetti R. F. da Silva P. A. Almeida-Reis R. Saraiva-Romanholo B. M. (2012). Rho-kinase inhibition attenuates airway responsiveness, inflammation, matrix remodeling, and oxidative stress activation induced by chronic inflammation. Am. J. Physiol. Lung Cell. Mol. Physiol. 11, L939–L952 10.1152/ajplung.00034.2012 23002076

[B87] Pouliot P. Olivier M. (2009). Opposing forces in asthma: regulation of signaling pathways by kinases and phosphatases. Crit. Rev. Immunol. 29, 419–442 10.1615/CritRevImmunol.v29.i5.40 20001889

[B88] Prado C. M. Leick-Maldonado E. A. Kasahara D. I. Capelozzi V. L. Martins M. A. Tibério I. F. L. C. (2005a). Effects of acute and chronic nitric oxide inhibition in an experimental model of chronic pulmonary allergic inflammation in guinea pigs. Am. J. Physiol. Lung Cell. Mol. Physiol. 289, L677–683 10.1152/ajplung.00010.2005 15937069

[B89] Prado C. M. Leick-Maldonado E. A. Arata V. Kasahara D. I. Martins M. A. Tibério I. F. L. C. (2005b). Neurokinins and inflammatory cell iNOS expression in guinea pigs with chronic allergic airway inflammation. Am. J. Physiol. 288, L741–748 10.1152/ajplung.00208.2004 15579630

[B90] Prado C. M. Leick-Maldonado E. A. Yano L. Leme A. S. Capelozzi V. L. Martins M. A. (2006). Effects of nitric oxide synthases in chronic allergic airway inflammation and remodeling. Am. J. Respir. Cell. Mol. Biol. 35, 457–465 10.1165/rcmb.2005-0391OC 16709960

[B91] Rocco P. R. Negri E. M. Kurtz P. M. Vasconcellos F. P. Silva G. H. Capelozzi V. L. (2001). Lung tissue mechanics and extracellular matrix remodeling in acute lung injury. Am. J. Respir. Crit. Care Med. 164, 1067–1071 10.1164/ajrccm.164.6.2007062 11587998

[B92] Rosenberg H. F. Dyer K. D. Foster P. S. (2013). Eosinophils: changing perspectives in health and disease. Nat. Rev. Immunol. 13, 9–22 10.1038/nri3341 23154224 PMC4357492

[B93] Ruiz-Schütz V. C. Drewiacki T. Nakashima A. S. Arantes-Costa F. M. Prado C. M. Kasahara D. I. (2009). Oral tolerance attenuates airway inflammation and remodeling in a model of chronic pulmonary allergic inflammation. Respir. Physiol. Neurobiol. 165, 13–21 10.1016/j.resp.2008.09.007 18930843

[B94] Russo M. Jancar S. Pereira de Siqueira A. L. Mengel J. Gomes E. Ficker S. M. (1998). Prevention of lung eosinophilic inflammation by oral tolerance. Immunol Lett. 61, 15–23 10.1016/S0165-2478(97)00155-7 9562371

[B95] Russo M. Nahori M. A. Lefort J. Gomes E. de Castro Keller A. Rodriguez D. (2001). Suppression of asthma-like responses in different mouse strains by oral tolerance. Am. J. Respir. Cell. Mol. Biol. 24, 518–526 10.1165/ajrcmb.24.5.4320 11350820

[B96] Sagara H. Yukawa T. Arima M. Makino S. (1993). Effects of capsaicin on the migration of eosinophils into the bronchi of guinea pigs. Aerugi 42, 236–242 8498895

[B97] Schaafsma D. Bos S. T. Zuidhof A. B. Zaagsma J. Meurs H. (2008). The inhaled Rho kinase inhibitor Y-27632 protects against allergen-induced acute bronchoconstriction, airway hyperresponsiveness, and inflammation. Am. J. Physiol. Lung Cell. Mol. Physiol. 295, L214–L219 10.1152/ajplung.00498.2007 18487358

[B98] Schaafsma D. Gosens R. Bos S. T. Meurs H. Zaagsma J. Nelemans A. (2004). Allergic sensitization enhances the contribution of Rho-kinase to airway smooth muscle contraction. Br. J. Pharmacol. 143, 477–484 10.1038/sj.bjp.0705903 15381630 PMC1575413

[B99] Schuiling M. Meurs H. Zuidhof A. B. Venema N. Zaagsma J. (1998). Dual action of iNOS-derived nitric oxide in allergy-induced airway hyperreactivity in conscious, unrestrained guinea pigs. Am. J. Respir. Crit. Care Med. 158, 1442–1449 10.1164/ajrccm.158.5.9803027 9817691

[B100] Schwartz N. Grossman A. Levy Y. Schwarz Y. (2012). Correlation between eosinophil count and methacholine challenge test in asymptomatic subjects. J. Asthma 49, 336–341 10.3109/02770903.2012.672613 22715867

[B101] Shamri R. Xenakis J. J. Spencer L. A. (2011). Eosinophils in innate immunity: an evolving story. Cell Tissue Res. 343, 57–83 10.1007/s00441-010-1049-6 21042920 PMC3679536

[B102] Sonar S. S. Ehmke M. Marsh L. M. Dietze J. Dudda J. C. Conrad M. L. (2012). Clara cells drive eosinophil accumulation in allergic asthma. Eur. Respir. J. 39, 429–438 10.1183/09031936.00197810 21828027

[B103] Spector S. L. Tan R. A. (2012). Is a single blood eosinophil count a reliable marker for “eosinophilic asthma?”. J. Asthma 49, 807–810 10.3109/02770903.2012.713428 22900679

[B104] Spencer L. A. Szela C. T. Perez S. A. Kirchhoffer C. L. Neves J. S. Radke A. L. (2009). Human eosinophils constitutively express multiple Th1, Th2, and immunoregulatory cytokines that are secreted rapidly and differentially. J. Leukoc. Biol. 85, 117–123 10.1189/jlb.0108058 18840671 PMC2626765

[B105] Starling C. M. Prado C. M. Leick-Maldonado E. A. Lanças T. Reis F. G. Aristóteles L. R. (2009). Inducible nitric oxide synthase inhibition attenuates lung tissue responsiveness and remodeling in a model of chronic pulmonary inflammation in guinea pigs. Respir. Physiol. Neurobiol. 165, 185–194 10.1016/j.resp.2008.11.011 19118648

[B106] Taki F. Kume H. Kobayashi T. Ohta H. Aratake H. Shimokata K. (2007). Effects of Rho-kinase inactivation on eosinophilia and hyper-reactivity in murine airways by allergen challenges. Clin. Exp. Allergy 37, 599–607 10.1111/j.1365-2222.2007.02693.x 17430358

[B107] Taylor E. L. Megson I. L. Haslett C. Rossi A. G. (2003). Nitric oxide: a key regulator of myeloid inflammatory cell apoptosis. Cell Death Differ. 10, 418–430 10.1038/sj.cdd.4401152 12719719

[B108] Tibério I. F. Leick-Maldonado E. A. Miyahara L. Kasahara D. I. Spilborghs G. M. Martins M. A. (2003). Effects of neurokinins on airway and alveolar eosinophil recruitment. Exp. Lung Res. 29, 165–177 10.1080/01902140303772 12637228

[B109] Tibério I. F. Turco G. M. Leick-Maldonado E. A. Sakae R. S. Paiva S. O. do Patrocinio M. (1997). Effects of neurokinin depletion on airway inflammation induced by chronic antigen exposure. Am. J. Respir. Crit. Care Med. 155, 1739–1747 10.1164/ajrccm.155.5.9154886 9154886

[B110] Trivedi S. G. Lloyd C. M. (2007). Eosinophils in the pathogenesis of allergic airways disease. Cell. Mol. Life Sci. 64, 1269–1289 10.1007/s00018-007-6527-y 17364144 PMC3383618

[B111] Uhm T. G. Kim B. S. Chung I. Y. (2012). Eosinophil development, regulation of eosinophil-specific genes, and role of eosinophils in the pathogenesis of asthma. Allergy Asthma Immunol. Res. 4, 68–79 10.4168/aair.2012.4.2.68 22379601 PMC3283796

[B112] Vaickus L. J. Bouchard J. Kim J. Natarajan S. Remick D. G. (2010). Oral tolerance inhibits pulmonary eosinophilia in a cockroach allergen induced model of asthma: a randomized laboratory study. Respir. Res. 11, 160 10.1186/1465-9921-11-160 21092270 PMC3016351

[B113] Venge P. (2010). The eosinophil and airway remodelling in asthma. Clin. Respir. J. 4, 15–19 10.1111/j.1752-699X.2010.00192.x 20500605

[B114] Vignola A. M. Kips J. Bousquet J. (2000). Tissue remodeling as a feature of persistent asthma. J. Allergy. Clin. Immunol. 105, 1041–1053 10.1016/S0091-6749(00)90053-8 10856134

[B115] Vignola A. M. Mirabella F. Costanzo G. Di Giorgi R. Gjomarkaj M. Bellia V. (2003). Airway remodeling in asthma. Chest 123, 417S–422S 10.1378/chest.123.3_suppl.417S-a 12629009

[B116] Walsh G. M. (2010). Targeting eosinophils in asthma: current and future state of cytokine- and chemokine-directed monoclonal therapy. Expert Rev. Clin. Immunol. 6, 701–704 10.1586/eci.10.58 20828276

[B117] Wardlaw A. J. (1999). Molecular basis for selective eosinophil trafficking in asthma: a multistep paradigm. J. Allergy. Clin. Immunol. 104, 917–926 10.1016/S0091-6749(99)70069-2 10550733

[B118] Watt A. P. Schock B. C. Ennis M. (2005). Neutrophils and eosinophils: clinical implications of their appearance, presence and disappearance in asthma and COPD. Curr. Drug Targets Inflamm. Allergy 4, 415–423 10.2174/1568010054526313 16101518

[B119] Weinstock J. V. Blum A. Walder J. Walder R. (1988). Eosinophils from granulomas in murine *Schistosomiasis mansoni* produce substance P. J. Immunol. 141, 961–966 2456338

[B120] Xisto D. G. Farias L. L. Ferreira H. C. Picanco M. R. Amitrano D. Lapa E. S. J. R. (2005). Lung parenchyma remodeling in a murine model of chronic allergic inflammation. Am. J. Respir. Crit. Care Med. 171, 829–837 10.1164/rccm.200408-997OC 15657464

[B121] Yoshii A. Iizuka K. Dobashi K. Horie T. Harada T. Nakazawa T. (1999). Relaxation of contracted rabbit tracheal and human bronchial smooth muscle by Y-27632 through inhibition of Ca^2+^ sensitization. Am. J. Respir. Cell. Mol. Biol. 20, 1190–1200 10.1165/ajrcmb.20.6.3441 10340938

[B122] Zhu M. Liu P. Y. Kasahara D. I. Williams A. S. Verbout N. G. Halayko A. J. (2011). Role of Rho kinase isoforms in murine allergic airway responses. Eur. Respir. J. 38, 841–850 10.1183/09031936.00125010 21565918 PMC3263528

